# A case report on the management of sepsis complicated by eroded mixed hemorrhoids following breast cancer chemotherapy

**DOI:** 10.1097/MD.0000000000050716

**Published:** 2026-09-25

**Authors:** Zi Ye, Hongmei Xie, Xiaoqian Lan, Qiuzhou Wang, Lan Fu

**Affiliations:** a Department of General Surgery, West China Hospital, Sichuan University, Chengdu, China; b Department of Breast Disease Center, West China Hospital, Sichuan University, Chengdu, China; c Department of West China School of Nursing, Sichuan University, Chengdu, China; d Department of Anesthesiology, West China Hospital, Sichuan University, Chengdu, China.

**Keywords:** breast cancer, case, chemotherapy, mixed hemorrhoids, septicemia

## Abstract

**Rationale:**

Sepsis is a life-threatening complication in breast cancer patients undergoing chemotherapy, particularly when compounded by anorectal conditions. The management of eroded mixed hemorrhoids in the setting of severe chemotherapy-induced neutropenia and septicemia presents a clinical dilemma, as standard surgical interventions carry prohibitive risks. This case report details a successful conservative, multimodal approach to this rare and critical scenario.

**Patient concerns:**

A 51-year-old female with stage IIIA human epidermal growth factor receptor 2-positive breast cancer developed a high fever (40.1°C) 1 week after her first cycle of neoadjuvant TCHP (docetaxel, carboplatin, trastuzumab, and pertuzumab) chemotherapy. She presented with severe bone marrow suppression and constipation, which led to the manual reduction of prolapsed hemorrhoids, resulting in an eroded, inflamed perianal mass.

**Diagnoses:**

The patient was diagnosed with septicemia (confirmed by *Escherichia coli* bacteremia), chemotherapy-induced grade IV bone marrow suppression (white blood cell count 0.17 × 10^9^/L, platelet count 6 × 10^9^/L), and mixed hemorrhoids with erosion and secondary perianal infection.

**Interventions:**

Given the high risk of surgery due to critical thrombocytopenia and neutropenia, a conservative strategy was implemented. This included broad-spectrum systemic antibiotics (meropenem, vancomycin, amikacin, amphotericin B sequentially), aggressive hematologic support (granulocyte colony-stimulating factor, blood transfusions), and meticulous multimodal local wound care. The local regimen comprised saline cleansing, infrared therapy, recombinant human epidermal growth factor gel, zinc oxide ointment, compound borneol ester suppositories, and the topical application of a self-prepared traditional Chinese medicine, “Liuhe Dan.”

**Outcomes:**

After 30 days of treatment, the patient became afebrile, and her perianal swelling and inflammation completely resolved. The eroded hemorrhoid healed entirely, allowing for a successful transfer to a lower-level hospital for continued supportive care.

**Lessons:**

This case demonstrates that in severely neutropenic and thrombocytopenic patients, a conservative, integrated approach combining systemic anti-infective therapy with advanced local wound care and traditional Chinese medicine can successfully manage life-threatening sepsis originating from eroded hemorrhoids. It underscores the critical importance of perianal hygiene, prompt management of chemotherapy-induced constipation and bone marrow suppression, and the potential of nonsurgical strategies in high-risk surgical candidates.

## 1. Introduction

Mixed hemorrhoids with erosion are a common anorectal condition characterized by the presence of both internal and external hemorrhoids, often leading to symptoms such as pain, bleeding, and discomfort.^[1,2]^ The incidence of mixed hemorrhoids is notably high, with estimates suggesting that up to 50% of the population may experience this issue at some point in their lives.^[3,4]^ In patients undergoing chemotherapy for breast cancer, the risk of developing mixed hemorrhoids may increase due to factors such as immunosuppression and alterations in bowel habits.^[5]^ The presence of erosive lesions can significantly exacerbate the patient’s condition, particularly in the context of septicemia, which can arise as a complication of chemotherapy. This combination poses serious risks, potentially leading to severe infections and further compromising the patient’s already fragile health status.

Currently, there are few reported cases of septicemia following breast cancer chemotherapy combined with mixed hemorrhoids and erosion. Surgical intervention is often considered the primary treatment for mixed hemorrhoids^[6]^; however, our approach emphasizes conservative management strategies. We implemented a regimen that included meticulous perianal skin care, oral lactulose for stool softening, infrared physical therapy, custom-made topical herbal applications, and the use of Ma Ying Long musk hemorrhoid ointment. Additionally, we applied recombinant human epidermal growth factor gel and zinc oxide ointment locally, along with compound carraghenates suppositories for rectal management. These measures effectively controlled the infection associated with mixed hemorrhoids and erosion, illustrating the potential for nonsurgical management in this complex clinical scenario.

## 2. Case report

A 51-year-old female patient was admitted with a chief complaint of fever lasting 2 days after being diagnosed with right breast cancer and undergoing chemotherapy. The patient had been diagnosed with invasive ductal carcinoma of the right breast, with a clinical stage of cT2N1M0 (Stage IIIA), and was confirmed to be estrogen receptor negative, progesterone receptor negative, and human epidermal growth factor receptor 2 positive. One week prior to admission, on April 12, she had received her first cycle of neoadjuvant chemotherapy with a TCHP regimen (docetaxel, carboplatin, trastuzumab, and pertuzumab). She did not receive prophylactic granulocyte colony-stimulating factor following this cycle, and had no prior adverse events from chemotherapy as this was her initial treatment. Following chemotherapy, she failed to receive timely treatment for leukocyte and platelet elevation. Additionally, she experienced constipation and resorted to manual defecation, which resulted in the prolapse of mixed hemorrhoids. Upon admission, her highest recorded temperature was 40.1°C. Laboratory tests revealed a white blood cell count of 0.17 × 10^9^/L, a platelet count of 6 × 10^9^/L, and a red blood cell count of 2.55 × 10^12^/L. Blood cultures indicated an infection with *Escherichia coli*. Physical examination showed a central anal region with a prominent rim, significant tenderness, and a dark purple, fluctuant mass in the perianal area, with subcutaneous ecchymosis and thrombosis. The skin temperature was elevated, accompanied by a small amount of yellow exudate (Fig. 1A). The final diagnoses included: septicemia; chemotherapy-induced grade IV bone marrow suppression; and mixed hemorrhoids with erosion. Given the severity of the patient’s condition, with septicemia likely stemming from an infection of the perianal mixed hemorrhoids, the medical team prioritized identifying the source of the infection while actively managing the local mixed hemorrhoids. The systemic treatment regimen included the administration of meropenem for infection control, total parenteral nutrition, and treatments aimed at elevating leukocyte and platelet counts, as well as lactulose for stool softening. Local treatment involved cleaning and disinfecting the perianal area with erythromycin, applying a self-prepared traditional Chinese medicine (TCM) “Liuhe Dan” externally to reduce swelling and alleviate pain, and using Ma Ying Long musk hemorrhoid ointment to promote blood circulation, reduce swelling, and facilitate tissue regeneration. Five days after admission, the patient continued to exhibit high fever; however, the swelling of the prolapsed internal hemorrhoids had improved, although blisters appeared on both sides of the prolapsed hemorrhoids (Fig. 1B, C). In response, the treatment plan was promptly adjusted to include vancomycin for further infection management while maintaining the same local treatment for the mixed hemorrhoids. Following these interventions, the patient’s overall condition gradually stabilized, and her symptoms significantly improved, resulting in notable therapeutic success.

**Figure 1.**
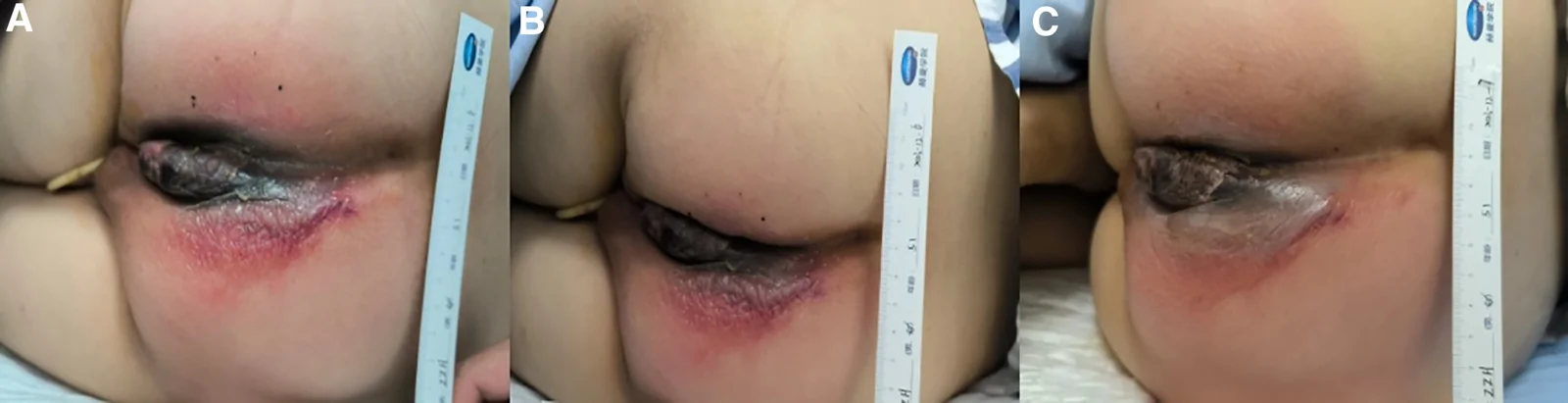
(A) Dark purple mixed hemorrhoids with inflammation. (B, C) Blisters appearing on both sides of the prolapsed internal hemorrhoids.

The antibiotic regimen was escalated sequentially: meropenem was initiated on admission (day 1), vancomycin was added on day 5 due to persistent fever, and amikacin with amphotericin B was added on day 10 when the patient remained febrile with ruptured perianal lesions. After 10 days of treatment, the patient continued to experience high fever and fatigue, with no improvement in systemic symptoms. The swelling of the prolapsed internal hemorrhoids had somewhat diminished; however, the blisters on both sides of the prolapsed internal hemorrhoids had ruptured, resulting in bloody exudate (Fig. 2A). The treatment plan was further adjusted, and the patient received a combined systemic antibiotic therapy of meropenem, amikacin, and amphotericin B. For local management of the mixed hemorrhoids, the anal region was gently cleaned with saline, followed by infrared light exposure to maintain cleanliness and dryness of the perianal skin. Subsequently, Human Epidermal Growth Factor Gel was applied locally, which promotes the synthesis of DNA, RNA, and hydroxyproline during the skin wound healing process, accelerates the formation of granulation tissue, and enhances the proliferation of epithelial cells, thereby shortening the healing time of the wound. Zinc oxide ointment was then reapplied to help constrict the skin, maintain local dryness, and promote the healing of small skin ulcers. Finally, a compound borneol ester suppository was administered rectally. This suppository has a lubricating effect, facilitating the ease of stool passage, and contains titanium dioxide and zinc oxide, which provide antipruritic effects, reduce congestion and inflammation of the anal canal and rectum, exert astringent effects, and promote healing. Granulation tissue began to form at the site of the perianal ulceration (Fig. 2B, C).

**Figure 2.**
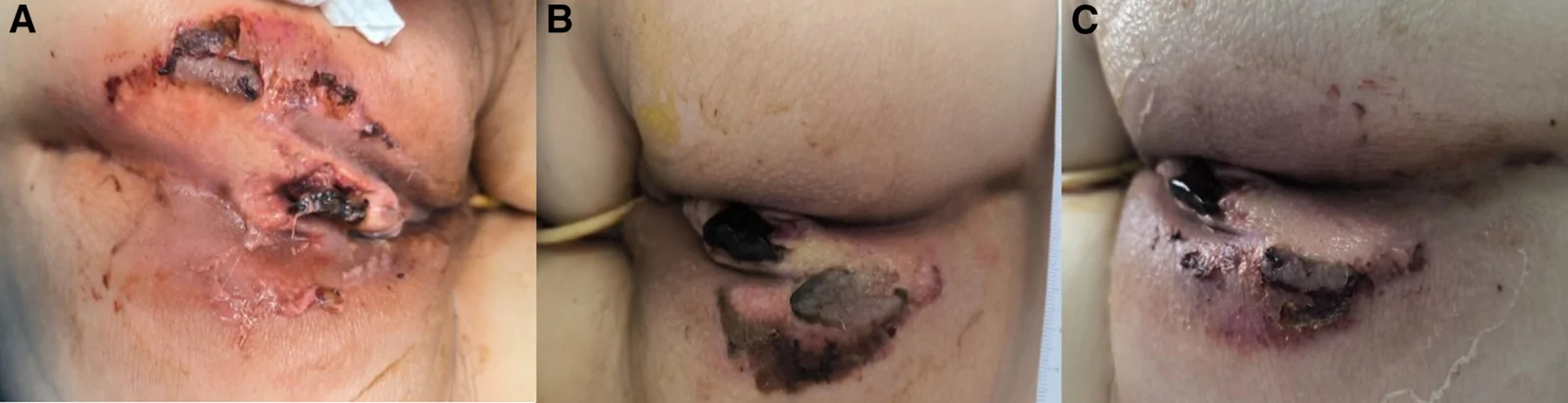
(A) Blisters on both sides of the prolapsed internal hemorrhoids have ruptured. (B, C) Granulation tissue is beginning to form at the sites of rupture.

After 20 days of treatment, with the control of inflammation, the granulation tissue at the perianal ulcer gradually matured. The surrounding epithelial cells began to migrate toward the wound, progressively covering the surface of the ulcer. The epithelial cells also resynthesized and repaired the basement membrane beneath the wound, gradually restoring the normal skin structure (Fig. 3). After 30 days of treatment, the patient was afebrile, there was no redness or swelling in the perianal area, and the external hemorrhoid that had ruptured had completely healed (Fig. 4).

**Figure 3.**
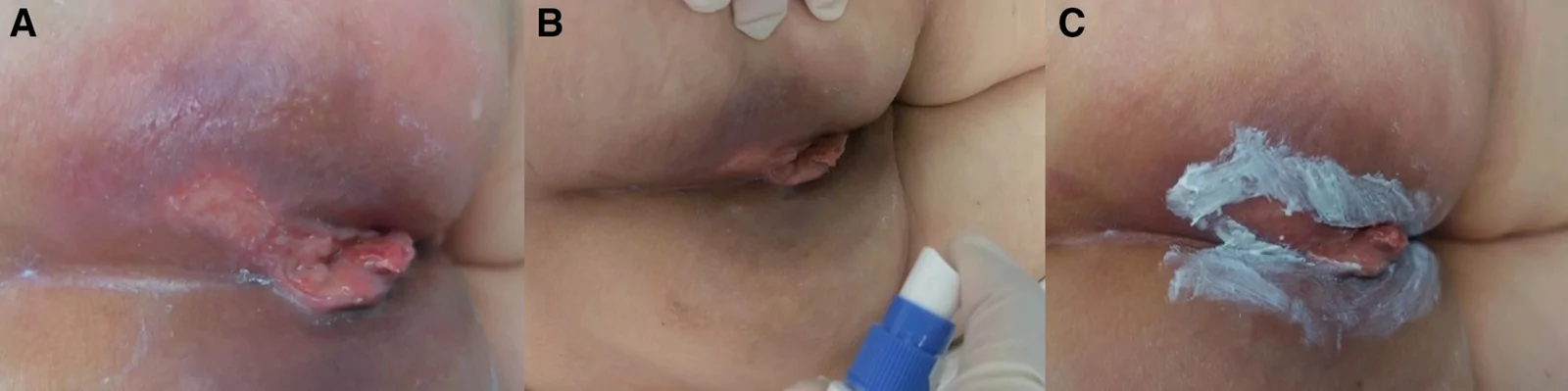
Progression of perianal wound healing at day 20 of treatment. (A) The swelling of the prolapsed internal hemorrhoids has significantly decreased. (B) The granulation tissue at the site of the previously ruptured blister has now matured and is covered by a superficial scab, indicating active epithelialization and wound contraction. (C) No active bleeding or purulent exudate is observed.

**Figure 4.**
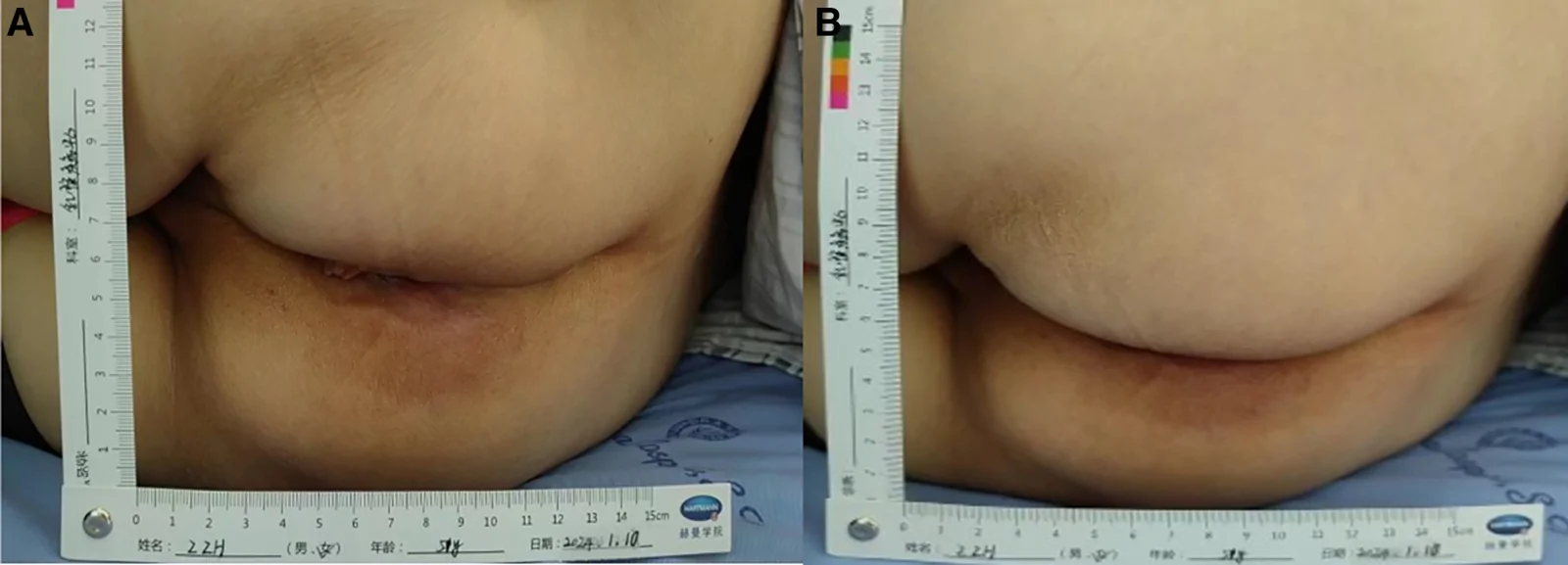
Complete healing of the perianal lesion at day 30 of treatment. (A) The perianal region shows no erythema, edema, or tenderness. The external hemorrhoid component, which was previously eroded, has healed completely with the formation of new, intact epithelium. (B) A closer view demonstrates the total resolution of the ulcerated area, with the skin regaining a normal appearance and texture, signifying successful conservative management.

## 3. Discussion

After breast cancer chemotherapy, patients may experience varying degrees of side effects such as bone marrow suppression, constipation, nausea, and vomiting.^[7]^ However, cases of constipation leading to hemorrhoid prolapse, perianal infection, and skin ulceration with bleeding are relatively rare. In this case, the patient experienced constipation at home after discharge and attempted to force a bowel movement, resulting in hemorrhoid prolapse. The prolapse was not treated further, leading to erosion of the mixed hemorrhoids, perianal skin infection, and ulceration with bleeding. Additionally, the patient did not receive the prescribed leukocyte-stimulating treatment, resulting in bone marrow suppression, which exacerbated the infection and led to sepsis, further compromising immune function and delaying healing of the perianal skin. This underscores the importance of proper follow-up and continuity of care for patients after chemotherapy, as well as enhancing patient compliance with medical advice.

Currently, the main treatment options for mixed hemorrhoids with erosion are surgical excision and laser therapy.^[8,9]^ In this case, considering that the patient was suffering from sepsis following breast cancer chemotherapy, complicated by mixed hemorrhoids with erosion and significant infection, we opted for a conservative treatment approach that combined systemic anti-infection therapy and local treatment. Rationale for conservative management: Surgical excision is the standard treatment for eroded hemorrhoids.^[8,9]^ However, in this patient, surgery was considered contraindicated due to life-threatening thrombocytopenia (platelet count 6 × 10^9^/L) and neutropenia (white blood cell count 0.17 × 10^9^/L), which placed her at extremely high risk of uncontrolled perioperative bleeding and fatal secondary infection. Additionally, her hemodynamic instability from sepsis precluded any elective surgical intervention. Therefore, we opted for a conservative approach combining systemic anti-infection therapy, aggressive hematologic support (granulocyte colony-stimulating factor and platelet transfusions), and meticulous local wound care. This strategy successfully controlled the infection and allowed for perianal wound healing without surgical morbidity. Initially, symptomatic treatments such as leukocyte-stimulating agents, blood transfusions, antibiotics, and nutritional support were administered to control the infection and enhance the patient’s immunity. Subsequently, we actively implemented a combination of traditional Chinese and Western medicine for local treatment, ensuring proper hygienic care. Western topical ointments were applied alongside a self-prepared TCM, “Liuhe Dan,” a topical herbal preparation developed by our institution. Liuhe Dan is composed of multiple botanical drugs including Rhei Radix et Rhizoma (Dahuang), Phellodendri Chinensis Cortex (Huangbai), Bletillae Rhizoma (Baiji), Menthae Haplocalycis Herba (Bohe), Angelicae Dahuricae Radix (Baizhi), smoked plum (Wumei), and several other components, which are powdered and mixed with honey into a paste.^[10]^ In our patient, approximately 10 g of the paste was applied to the perianal lesion once daily, spread to a thickness of about 6 mm and extending approximately 1 cm beyond the margin of the edema, then covered with sterile gauze and secured with tape. This formula was originally developed by a renowned TCM practitioner at our institution and has been clinically used for acute incarcerated hemorrhoid edema and postoperative edema following mixed hemorrhoid surgery.^[10]^ Mechanistically, Liuhe Dan is formulated based on the principles of clearing heat, removing toxicity, promoting blood circulation, and resolving stasis. Experimental studies have demonstrated that topical application of Liuhe Dan can significantly reduce the expression of inflammatory cytokines such as IL-1β and TNF-α in infected wounds, thereby limiting the release of inflammatory mediators and creating favorable conditions for wound healing. A previous clinical study demonstrated that external application of Liuhe Dan significantly reduced anal edge edema scores and wound pain scores, while also shortening hospital stay and wound healing time in patients with mixed hemorrhoids.^[11]^ In the present case, we employed Liuhe Dan as an adjunctive therapy to control local inflammation and promote the reduction and retraction of the hemorrhoids. Additionally, physical infrared therapy was utilized to avoid perianal moisture and promote skin scabbing. Sepsis following chemotherapy poses a severe threat to the patient’s life and prognosis. While surgical excision can rapidly remove the lesions, it may increase the patient’s pain and risk of reinfection. In contrast, our conservative local treatment effectively controlled the local inflammation of the mixed hemorrhoids and facilitated their reduction and retraction. Research has shown that^[12,13]^ rubber band ligation and sclerotherapy can reduce blood flow to internal hemorrhoids, alleviating symptoms. The therapeutic effects of these methods on patients with erosive mixed hemorrhoids merit further investigation in future studies.

The treatment of cases involving sepsis following breast cancer chemotherapy, complicated by mixed hemorrhoids with erosion, is relatively rare in the literature. In this case, the patient received systemic anti-infection therapy in conjunction with a combination of traditional Chinese and Western medicine for local treatment. This included the active administration of antibiotics, blood transfusions, and other symptomatic management; meticulous care of the perianal skin; and the application of infrared lamp therapy to maintain cleanliness and dryness of the anal area. Subsequently, topical applications of TCM “Liuhe Dan,” Human Epidermal Growth Factor Gel, and zinc oxide ointment were employed, along with the use of a compound of butyrate ester suppositories to alleviate congestion and inflammation in the anal canal and rectum, reduce swelling, and promote the healing of ulcerated areas. After approximately 30 days of treatment, the patient exhibited no signs of redness or swelling in the perianal region, and the external hemorrhoid erosion had completely healed, enabling a successful discharge. Establishing the infectious source: in this case, the source of sepsis was presumed to be the eroded mixed hemorrhoids based on several clinical findings. First, blood cultures identified *E coli*, a pathogen commonly associated with perianal and gastrointestinal infections. Second, the patient had a visible, inflamed, and eroded perianal lesion with purulent exudate at the time of fever onset, with no other identifiable infectious foci on physical examination or imaging. Third, the patient’s severe neutropenia (white blood cell count 0.17 × 10^9^/L) and thrombocytopenia (platelet count 6 × 10^9^/L) following chemotherapy created a vulnerable state in which a local perianal infection could readily disseminate systemically. While a direct causal link cannot be definitively proven without tissue biopsy or additional microbiological sampling from the perianal lesion, the clinical timeline and absence of alternative infection sources strongly support this association.

It is important to acknowledge the significant limitations of this case report. The primary limitation is that the favorable local outcomes were achieved through a complex, multi-component treatment regimen, which makes it impossible to determine the specific contribution of any single intervention, including the TCM preparation “Liuhe Dan,” to the overall success. Although our use of Liuhe Dan is supported by a clinical study demonstrating its efficacy in reducing anal edema and pain in hemorrhoid patients,^[11]^ this evidence is derived from a single-center study and should be considered preliminary. Therefore, our findings should be interpreted as a single anecdotal experience that may not be generalizable to the broader population. Future prospective studies with larger sample sizes and rigorous designs are necessary to systematically evaluate the efficacy and safety of each component in this integrative approach. This case provides a reference for the conservative treatment of similar patients in the future.

## Author contributions

**Data curation:** Hongmei Xie.

**Investigation:** Xiaoqian Lan.

**Supervision:** Qiuzhou Wang.

**Writing – original draft:** Zi Ye.

**Writing – review & editing:** Lan Fu.
